# Supplementary material for: MetaRibo-Seq measures translation in microbiomes
Source: Nat Commun. 2020 Jun 29;11:3268. doi: 10.1038/s41467-020-17081-z (PMC7324362; doi:10.1038/s41467-020-17081-z)
Supplement: Supplementary file 10 — Supplementary Data 7 [file 41467_2020_17081_MOESM10_ESM.zip › File2/Confidence_High_Taxonomy/320423_out.krona.html]

Javascript must be enabled to view this page.

members
magnitude
magnitudeUnassigned
count
unassigned
taxon
rank

320423\_out

66

superkingdom
2
66

phylum
1239
66

186801
66
class

186802
66
order

family
186803
66

1
830
genus

species
1
45851

SRS063190\_contig\_number\_4505

species

SRS012969\_contig\_number\_25264SRS013158\_contig\_number\_contig-100\_154.42660SRS014235\_contig\_number\_45103SRS014613\_contig\_number\_5042SRS014736\_contig\_number\_1419SRS014948\_contig\_number\_24343SRS015190\_contig\_number\_16524SRS015217\_contig\_number\_8029SRS015794\_contig\_number\_12527SRS016018\_contig\_number\_14758SRS017191\_contig\_number\_10453SRS017433\_contig\_number\_17883SRS018427\_contig\_number\_contig-100\_562.186726SRS019267\_contig\_number\_contig-100\_307.63530SRS019601\_contig\_number\_2273SRS022137\_contig\_number\_21385SRS023346\_contig\_number\_contig-100\_501.60626SRS023526\_contig\_number\_22936SRS024492\_contig\_number\_7863SRS042628\_contig\_number\_29840SRS043411\_contig\_number\_17578SRS043667\_contig\_number\_7029SRS043701\_contig\_number\_16135SRS043768\_contig\_number\_32912SRS047433\_contig\_number\_20225SRS048164\_contig\_number\_32370SRS049959\_contig\_number\_24372SRS050026\_contig\_number\_11311SRS051610\_contig\_number\_9706SRS053335\_contig\_number\_contig-100\_202.104681SRS055982\_contig\_number\_18282SRS057478\_contig\_number\_9019SRS058723\_contig\_number\_contig-100\_701.63936SRS063370\_contig\_number\_13393SRS075773\_contig\_number\_33969SRS076804\_contig\_number\_12507SRS077294\_contig\_number\_19464SRS078176\_contig\_number\_23945SRS104084\_contig\_number\_contig-100\_548.90874SRS1041090\_contig\_number\_7583SRS1041091\_contig\_number\_21168SRS1041133\_contig\_number\_27098SRS1041136\_contig\_number\_26010SRS1041144\_contig\_number\_8749SRS104311\_contig\_number\_1555SRS104327\_contig\_number\_contig-100\_34.54302SRS104485\_contig\_number\_827SRS1055049\_contig\_number\_10859SRS1055056\_contig\_number\_13221SRS143342\_contig\_number\_19968SRS144537\_contig\_number\_contig-100\_552.250874SRS146764\_contig\_number\_24265SRS147346\_contig\_number\_54894SRS147377\_contig\_number\_contig-100\_560.80581SRS147425\_contig\_number\_contig-100\_395.82425SRS147614\_contig\_number\_2236SRS147766\_contig\_number\_13225SRS148253\_contig\_number\_580SRS148721\_contig\_number\_42918SRS148817\_contig\_number\_14153SRS149784\_contig\_number\_23757SRS893252\_contig\_number\_19670SRS893295\_contig\_number\_contig-100\_656.83280SRS893373\_contig\_number\_26326SRS893378\_contig\_number\_19102
39491
65
